# Metagenomics: aid to combat antimicrobial resistance in diarrhea

**DOI:** 10.1186/s13099-019-0331-8

**Published:** 2019-10-14

**Authors:** Rituparna De

**Affiliations:** Division of Bacteriology, National Institute of Cholera and Enteric Diseases, Kolkata, India

**Keywords:** Antimicrobial resistance, Antimicrobial resistance genes, Diarrhea, Gut, Pathogen, Microbiome, Commensal, Metagenomics, Probiotics

## Abstract

Antimicrobial resistance (AMR) has emerged as an obstacle in the supple administration of antimicrobial agents to critical diarrheal patients. Most diarrheal pathogens have developed resistance against the major classes of antibiotics commonly used for assuaging diarrheal symptoms. Antimicrobial resistance develops when pathogens acquire antimicrobial resistance genes (ARGs) through genetic recombination from commensals and pathogens. These are the constituents of the complex microbiota in all ecological niches. The recombination events may occur in the environment or in the gut. Containment of AMR can be achieved through a complete understanding of the complex and diverse structure and function of the microbiota. Its taxonomic entities serve as focal points for the dissemination of antimicrobial resistance genetic determinants. Molecular methods complemented with culture-based diagnostics have been historically implemented to document these natural events. However, the advent of next-generation sequencing has revolutionized the field of molecular epidemiology. It has revolutionized the method of addressing relevant problems like diagnosis and surveillance of infectious diseases and the issue of antimicrobial resistance. Metagenomics is one such next-generation technique that has proved to be a monumental advancement in the area of molecular taxonomy. Current understanding of structure, function and dysbiosis of microbiota associated with antimicrobial resistance was realized due to its conception. This review describes the major milestones achieved due to the advent and implementation of this new technique in the context of antimicrobial resistance. These achievements span a wide panorama from the discovery of novel microorganisms to invention of translational value.

## Introduction

AMR is adversely affecting the antimicrobial treatment regime in infectious diseases. It has emerged as a serious threat to human, animal and environmental health, healthcare systems and global economy [1]. It has been predicted that by 2050 100% drug-resistant infections will claim 10.2 million working-age lives per year if the AMR crisis is not reverted [1]. The problem has spread among gram-positive and gram negative bacteria. Multi-drug resistant *Staphylococcus aureus*, *Enterococcus* sp., *Mycobacterium tuberculosis*, *Pseudomonas* sp., *Acinetobacter* sp., *Salmonella enterica*, *Streptococcus pneumoniae*, *Klebsiella pneumoniae*, third-generation cephalosporin resistant *Neisseria gonorrhoeae* have emerged as critical threats [2, 3]. The misuse, overuse and the application of sub-lethal doses of antibiotics in farms, aquaculture, animal husbandry which initiated a farm-to-fork transmission of contaminating antibiotics, and also in clinical and veterinary practice, leakage of antibiotics into the natural environment preparing the ground for selection of antibiotic resistant bacteria are few of the multiple causes of AMR [4–6]. In developing countries, the major stimuli for AMR are lack of surveillance of AMR, poor quality of antibiotics, lack of regulatory guidelines and control on the sale and use of antibiotics resulting in the ease of their availability and clinical misuse often leading to prescription of wrong antimicrobials due to poor quality diagnosis and also administration of antimicrobials to patients even when not required [4]. These practices have resulted in critical AMR challenge in usage of fluoroquinolones, macrolides, third-generation cephalosporins and carbapenems and other classes of antibiotics in humans [2]. The major concern in agricultural and veterinary practice includes the medication of animals with antimicrobials that are used to treat infections in humans, pollution of the aquatic ecosystem from farm spillage and inadequate treatment of wastewater leading to incomplete breakdown of the antimicrobial compounds [6, 7]. Severe resistance against the major antibiotics including colistin and third-generation cephalosporins considered to be critical prophylactic agents has emerged among food-producing animals [7]. Fervent implementations are ongoing to mutilate the threat and curb the consequences. Antimicrobial resistance surveillance programs like NARMS and One Health Surveillance are the urgent need of the hour [8, 9]. Regulation of over-the-counter sale of drugs and antibiotic consumption can impede the growing threat of AMR progress. Research for finding alternative and effective drugs requires strong attention as AMR against available major classes of antibiotics has resulted as an aftermath of radical anthropogenic usage of these common drugs. Bacteria develop resistance when they acquire ARGs from related or distant species by horizontal gene transfer (HGT). Most of these genes are borne on mobile genetic elements (MGEs) like plasmids, integrons and transposons. The genetic recombination mechanisms helping in HGT and playing the pivotal role in the development of AMR among different bacterial species include conjugation, transduction and transformation. In addition, other mechanisms contributing to bacterial resistance to antibiotics include mutation in cellular genes, physical alteration or enzymatic degradation of the antibiotic by bacterial enzymes, active efflux of the antibiotic with the help of bacterial efflux proteins, mutation of the active target site of antimicrobial action, or a decrease in cell wall permeability. ARGs may originate in the environment or in the gut ecosystem [10–16]. It is often not just a single organism contributing to the origin and transmission of ARGs but a complex microbial community at play. The evolution of the concept of microbiome has established the understanding that outcome of disease, disease progression, antimicrobial resistance are all regulated by the microbiome. Most of the ARGs have their roots in commensals that form an integral part of the microbiome [17, 18] and contribute to the antimicrobial resistome [19].

## Metagenomics and AMR

Metagenomics has helped to elucidate a strong correlation between AMR and microbiome through the discovery of complex microbial communities and their functional components involved in AMR in bacteria [20]. At present almost all bacterial pathogens associated with infectious diseases have developed AMR.

Metagenomics developed as a standard typing method in order to overcome the obstacle imposed by standard culture methods to detect uncultivable or culture-resistant microorganisms. This had spread a thick shroud over our knowledge of bacterial diversity, for decades when laboratory-based culture methods were the sole methods of identification of bacteria from clinical and environmental samples. Metagenomics employs two distinct approaches for analysis: sequence-driven and function-driven [21]. Both are based on next-generation sequencing techniques and have been developed by a number of commercial organizations and operated on different platforms like the Illumina/Solexa,454/Roche, Ion PGM from Ion Torrent, AB SOLiD System, Oxford Nanopore MinION. These systems use different chemistry for their operation [22]. The technology has advanced in leaps and bounds with the refinement of bioinformatics platform and upgradation of hardware since its advent almost two decades ago.

Illumina/Solexa platform workflow operates on sequencing by synthesis (SBS) technology while 454/Roche platform uses pyrosequencing technique. Recent advancements like the availability of paired-end (PE) sequencing has enhanced efficiency and accuracy of data output of Illumina/Solexa platform by generating double the number of sequence reads for the same time during library preparation, detecting indels and elimination of artifacts [23].

In the sequence-driven method multiple sequence reads are generated and analyzed using sequence-analysis software. Direct DNA extraction from the clinical or environmental samples following standard optimized protocols to maximize coverage of bacterial diversity [24] and restriction digestion or mechanical shearing of the DNA followed by cloning into appropriate vectors to generate a metagenomic library is a crucial step before generating PCR amplicons for screening the library. Usually libraries are screened using PCR primers targeting conserved and ubiquitously present genes, most commonly, the 16 s ribosomal gene. Klindworth et al. designed a widely applicable primer set targeting the variable regions V3–V4 of the 16S rDNA which enabled the group to screen phylum spectrum in Archae and Bacteria maximally [25].

Shot-gun whole-genome sequencing is an alternative method for studying microbial community structural and functional diversity in which metabolic pathways and different genes can be screened and even near-complete whole-genomes of bacteria can be constructed from random sequence reads or contigs and is specially significant for metagenomic analysis of viral communities which lack 16S gene and its analogues [26]. The workflow typically includes sample collection, DNA extraction, library preparation, sequencing libraries and binning, assembly of contigs, and in silico analysis of the meta data. Library preparation, depending on sample type, may leverage an amplification step. According to some authors, this method is superior to the 16S rDNA amplicon sequencing as it demonstrates keener sensitivity and ability to detect higher species abundance [27, 28]. Venter et al. used the whole-genome shotgun sequencing to study microbial diversity in the Sargasso Sea and led the discovery of 1.2 million new genes and 782 new rhodopsin-like photoreceptors [29].

The function-driven analysis employs a different approach. Metagenomic libraries are screened for traits of interest by studying expression of those traits in transformed clones by subjecting these to sequencing and biochemical analysis. Functional metagenomics has been instrumental in characterizing antibiotic resistomes in different habitats [30]. Identification of antibiotics, antibiotic resistance genes, consensus and non-coding sequences associated with ARGs and MGEs are part of functional metagenomics [31]. The first metagenomically identified antibiotic turbomycin A and B, were discovered by this method [32]. However, functional metagenomics occasionally fails to detect antibiotic resistance that results from mutations of antibiotic targets [30].

Sequence-driven analysis and functional metagenomics have been applied hand in hand culminating in monumental discoveries. Rondon et al. constructed a metagenomic library using a BAC (Bacterial Artificial Chromosome) vector from DNA isolated from the soil. They used it to study phylogenetic diversity of soil microorganisms and also to identify and characterize enzymes and other phenotypes. Their work demonstrated the potential of metagenomics to identify genetic traits from uncultured microorganisms [33]. Recent advancements in annotation methods like the development of poreFUME pipeline has benefitted analysis and profiling of resistome from functional metagenomic data [34].

## Microbiome: source and solution of AMR

**Fig. 1:**
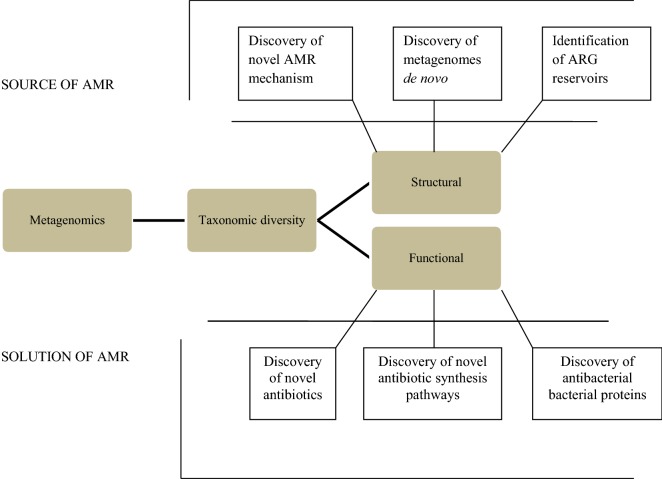
Implementation of metagenomics has helped in the current understanding of source and solution to the AMR challenge

The microbiome has emerged to be a key player in the emergence of AMR [20]. The members of these complex microbial communities in humans, animals and environment are reservoirs of ARGs [20] and serve as vital melting pots for the discovery of new ARGs [20, 35]. This is of immense epidemiological significance as new ARGs are the starting point of new problems in the AMR domain. Harnessing the high-throughput potential of sequence-based and functional metagenomics along with advancement in computational methods cataloguing of resistomes from diverse habitats has been achieved [30]. The next section describes how metagenomics has benefitted the study of the microbiome in the context of AMR (Fig. 1).

### Identification of ARGs and insight into gut resistome development

Metagenomic screening of gut resistome has helped in the identification of antibiotic resistance profile in human infants and adults and provided a sound understanding of the resistome development of the gut. In infants, a study showed that with the help of a combination of metagenomic approaches that included PCR-based analysis of fosmid clones and uncloned metagenomic DNA, which generated a library of ~ 220 Mb contained 0.45 ampicillin resistant hits/Mb and 0.059 gentamicin resistant hits/Mb, that infant gut resistome is replete with ARGs against aminoglycosides and β-lactam antibiotics also showing that the infant gut resistome establishes even before exposure to antibiotics, may be even at birth. The resistome profile could be associated with common gut bacteria like *Eschericia coli*, *Enterococcus* sp., *Clostridium difficile* and also to cryptic environmental bacteria [36] showing the importance of sequence-based and functional metagenomics to be pursued together. Another study focused on the pediatric (infant and children) microbiome and was conducted using the Illumina HiSeq 2000 platform. The PARFuMS computational pipeline was employed for raw data assembly and annotation. The study showed that the pooled DNA from fecal samples of healthy infants and children without recent antibiotic exposure harbored ARGs against 14 out of 18 tested potential antimicrobials belonging to 8 classes of antibiotics and the sequences identified included chloramphenicol acetyltransferases, drug-resistant dihydrofolate reductases, rRNA methyltransferases, transcriptional regulators, multidrug efflux pumps, and all major classes of beta-lactamase, aminoglycoside-modifying enzyme, and tetracycline resistance protein. Some of these elements were borne on MGEs and some indicated cryptic organisms as their source raising high caution on AMR [37].

The transmission of resistome from mother to infant has been scrutinized by several researchers. These studies showed that resistome acquisition of the infant may be pre-partum, intra-partum and post-partum not only through breast-milk and from other body sites of the mother [38] but is also strongly influenced by the exposure of the infant to multi-drug resistant organisms [39]. Ghosh et al. showed that AMR in infants may not be maternally transmitted but develops with environmental exposure to antibiotic resistant organisms [39].

### Resistome profile: identity signature

Resistome of individuals vary with age and geographical location [39, 40]. It is influenced by factors like diet, lifestyle, environmental contaminants, socio-economic and antibiotic usage practices which also determine the microbiome composition of the human gut [41–44]. Therefore microbiome and resistome profiles can be strongly correlated [20]. Resistome profile can be used as an identity marker to identify the country of origin of individuals as it is strongly associated with country-specific social and medical practices [45].

Ghosh et al. screened for antibiotic resistance determinants against 240 different antibiotics in previously characterized 275 gut metagenomes corresponding to 275 individuals from eight nationalities (Indian, American, Danish, French, Italian, Spanish, Japanese and Chinese). They detected ARGs against 53 antibiotics. The findings conformed to geography/country-specific patterns. The gut microbiome signatures could be aligned under four distinct resistotypes, based on their ARG profile. According to this in silico analysis resistance to tetracycline (267/275), bacitracin (263/275) and vancomycin (255/275) were the highest. Source of the ARGs was also successfully traced. For example tetracycline resistance emanated mainly from the phylum *Bacteroidetes* and the family *Clostridiaceae* [39].

Forslund et al. conducted metagenomic analysis of resistome profiles on 207 fecal samples from three different countries, USA, Spain and Denmark. 252 metagenomes generated were screened for 380 antibiotic resistance determinants covering 68 classes of antibiotics and their sub-classes through the ARDB database. Determinants against 50 classes and sub-classes of antibiotics were detected. A strong correlation between ARG patterns existed with the usage of their targets in animal husbandry and the amount of exposure time to these antibiotics. Results were strongly associated with commercial and clinical antibiotic usage practices varying from country to country [40].

Hendriksen et al. conducted a very significant study encompassing metagenomic analysis of urban domestic sewage samples from 79 locations covering 7 geographical regions from 74 cities in 60 countries using Illumina Hi-Seq platform and the resulting data generated was processed using MGmapper and reported that AMR profile strongly correlates with socio-economic, health and environmental factors of a country and a stronger regional difference was observed at the AMR class level than ARG level. The dominant bacterial genera detected were *Faecalibacterium*, *Bacteroides*, *Escherichia*, *Streptococcus* and *Bifidobacterium*, which are predominantly, fecal. Other highly abundant bacterial genera detected were *Acidovorax* and *Acinetobacter*, which are most likely environmental bacterial genera. A total of 1625 different ARGs belonging to 408 gene groups were detected and included CTX-M, NDM, *mcr* and *optrA* which have emerged recently. ARGs against macrolides, tetracyclines, aminoglycosides, beta-lactams and sulfonamides were the most abundant [46].

### Discovery of new ARGs

Functional metagenomics has helped in the discovery of new antimicrobial resistant determinants and mobilome. This helped in revealing novel mechanisms of antibiotic resistance [20, 37]. Functional metagenomics is particularly useful for the identification of new ARGs in natural environments as the method overlooks the compulsion of having previous knowledge of these genes [47]. The functional metagenomics approach can be used to obtain a sea of information regarding antibiotic resistance mechanisms particularly in highly heterogenous and diverse natural environments like the soil [47]. Antibiotic biosynthesis clusters can be examined for ARGs which provide self resistance to the antibiotic producers by various mechanisms [47].

Forsberg et al. studied the relationship between 18 antibiotics and 18 agricultural and grassland soils using functional metagenomics and discovered 2895 novel ARGs associated with every known antimicrobial resistance mechanism [20].

A functional metagenomics study by Moore et al. led to the discovery of three novel resistance genes, namely a 16S rRNA methylase conferring aminoglycoside resistance, and two tetracycline-resistance proteins nearly identical to a bifidobacterial MFS transporter (*B. longum S. longum* JDM301) [37].

Advancements in the computational pipeline like development of methods like fARGene for the identification and reconstruction of ARGs directly from shotgun-sequencing data has helped in the highly accurate identification of novel ARGs without the help of available reference and high-quality assembly from metagenomic fragments with low identity to previously known ARGs, completely based on optimized probabilistic gene models [48]. Such discovery is a great stride towards finding solutions for AMR as they will lead to the discovery of novel ARGs without any logistic impediment.

### Identification of environmental reservoirs of ARGs

Functional metagenomics has helped in identification of potential reservoirs of antimicrobial resistance determinants. These are centres of transmission of ARGs [37]. Identification of reservoirs of ARGs is foremost in the measures taken to solve AMR crisis. Environmental metagenomes explicitly vary from one another in microbiota composition and resistome pattern [49]. Metagenomics helped to understand which environmental niche may primarily be a source of dissemination of which type of antimicrobial resistance mechanism [50].

A study on the healthy infant gut resistome showed that it harbors a rich diversity of clinically relevant ARGs even without prior exposure to the selection pressure of antibiotics indicating the involvement of cryptic gut microbes as important sources of ARGs which are transferable. The study emphasized the importance of the healthy human gut as a reservoir of ARGs [37]. Forsberg et al. used functional metagenomics along with a pipeline for the de novo assembly of short-read sequence data from metagenomic data (PARFuMS), on a collection of 95 soil-derived cultures of bacteria highly resistant to various antibiotics and that were derived from 11 U.S soil and provided evidence for recent exchange of ARGs between environmental bacteria and clinical pathogens. The study identified multidrug-resistant soil bacteria harboring resistance gene cassettes against five classes of antibiotics (β-lactams, aminoglycosides, amphenicols, sulfonamides, and tetracyclines) with nucleotide similarity with human pathogens in noncoding regions and sequences associated with mobilome, providing evidence of lateral exchange of ARGs serving as an important agent of antibiotic resistance transmission between humans and environment [51]. Fitzpatrick and Walsh used environmental shotgun sequencing metagenomic datasets downloaded from the MG-RAST server to study and compare the distribution and relative abundance of clinically significant ARGs across diverse environmental (soil, water, plant, animal, insect) and human metagenomes and concluded that the human and mammalian gut harbors the highest diversity of clinically significant ARGs. The authors found that ARGs are ubiquitously distributed with varying relative abundance across all environmental niches and occur in commensals, pathogens, phytobacteria and environmental bacteria [50]. Another study showed that environmental metagenomes were characterized by high taxonomic diversity and high diversity of biocide/metal resistance genes and MGEs which is just the contrary for human and animal metagenomes which have high relative abundance of ARGs [49]. Tyagi et al. studied the fish metagenome and found a high prevalence of pathogenic bacteria (three-fourth of gut microbiota mapping to phylum Proteobacteria), low abundance of probiotic bacteria and 51 different types of ARGs belonging to 15 AMR gene families associated with resistance against 24 antibiotic types. Some of these ARGs were located on sequences of plasmid origin foreboding transmissibility of ARGs. The existence of MGEs carrying ARGs and pathogens in the fish gut is highly alarming and predictive of the evident genetic exchange of ARGs between different bacterial phyla making the fish gut a potential reservoir of ARGs and a vulnerable epicenter of HGT [52].

### Discovery of new antibiotics

Metagenomics has helped to discover an entire uncharted territory of unknown microbes and their potential role in AMR. At the same time, it has helped to discover new natural antibiotics and antibiotic biosynthesis pathways. Compared to anticancer drug development, development of new antimicrobials had warranted less attention over the last decade [47]. Metagenomics has helped in generating enormous information on microbial metabolism leading to new antimicrobial discovery [53]. Tyagi et al. recently studied the gut metagenome of the freshwater carp *Labeo rohita* using shotgun metagenomic sequencing and came across a plethora of genomic sequences which might be associated with synthesis of natural antibiotics which could become promising alternative drugs and would alleviate the problem of AMR [52].

## Metagenomics: a timely solution to the AMR challenge in diarrhea

Diarrheal illness continues to be a major problem in developing countries like India with a large section of the population languishing in poverty and lacking access to uncontaminated water and sanitation and devoid of hygienic living environment, lacking education and awareness about the outcome of exposure to contaminated water and environment. A similar scenario exists in many other countries of Asia and Africa [54, 55]. Diarrhea is responsible for 2.2 million deaths globally every year and is the second leading cause of death and malnutrition in children less than 5 years old [https://www.who.int/topics/diarrhoea/en]. AMR rate in diarrheal pathogens is high and has aggrieved the management of diarrheal diseases [56]. Centers for Disease Control and Prevention (CDC) in the US, estimated that 23,000 people die from antimicrobial resistant infections among 2.0 million people who contract it [57]. The CDC has categorized 18 MDR pathogens as urgent, serious and concerning threats. This emphasizes the gravity of the AMR threat in the domain of infectious diseases. Most of these are diarrheal pathogens and are causes of potent enteric infections and GI tract illness. *Clostridium difficile,* Carbapenem—resistant *Enterobacteriaceae* (CRE)*, Campylobacter* sp., Extended-spectrum Beta-lactamase (ESBL) producing *Enterobacteriaceae,* Drug-resistant non-typhoidal *Salmonella*, Drug-resistant *Shigella* appear in the list [58].

*Campylobacter* sp. has developed resistance towards the major antibiotics recommended for treatment of diarrhea, including ciprofloxacin, which is the first empirical treatment for diarrhea. Other drugs that fail to be functional in *Campylobacter* infections include ampicillin, erythromycin, tetracycline and trimethoprim-sulfamethoxazole [59]. Non-typhoidal *Salmonella* sp. are resistant to ampicillin, ciprofloxacin and fluoroquinolones [59]. Extended-spectrum β-lactamase-producing *Enterobacteriaceae* have been reported in a number of surveillance studies. These are resistant to penicillin derivatives, folate pathway inhibitors, fluoroquinolones, third-generation cephalosporins [60, 61].

Health-care researchers have developed probiotics to administer diarrheal treatment. Probiotics are used to prevent diarrhea and also as supplement with antibiotics or alone to make diarrheal treatment effective [62, 63]. These are also being developed as agents for the prevention of antimicrobial resistance [64] which is an enormous stride towards solving the AMR problem. The effect of probiotics on gut microbiota has been successfully studied with metagenomics to assess the functional efficacy of probiotics to minimize symptoms associated with diarrhea in experimental animals and could soon find its way into development for clinical applications [65].

Metagenomics has helped in undertaking large-scale epidemiological screening studies to gain complete knowledge of AMR profile of enteric pathogens [66]. It has contributed to the understanding of diarrheal etiology dissemination of AMR in the community and between the environment and community [66]. Kumar et al., studied the AMR pattern in 654 different enteric pathogens spanning 8 genera (*Escherichia, Shigella, Klebsiella, Salmonella, Providencia, Vibrio, Pseudomonas, Aeromonas*) and isolated in two different parts of India, primarily from cases of acute diarrhea with respect to 22 different antibiotics belonging to 9 classes. The results revealed that pathogens were multi-drug resistant and that resistance determinants borne on MGEs have potential threat of dissemination into the community. The study was performed using metagenomic techniques and included complete whole genome sequence analysis of 6 extensive drug-resistant (XDR) enteric pathogens belonging to 6 distinct genera. The study enabled one to understand AMR pattern of enteric pathogens in India and how it changes temporally and spatially [66]. Bag et al. characterized five commensal enteric bacteria that are the dominant residents of the healthy Indian gut and from their antimicrobial resistance phenotypic and genotypic characteristics concluded that they act as potential antibiotic resistance reservoirs and play a role in dissemination of ARGs to enteric pathogens [18].

**Fig. 2:**
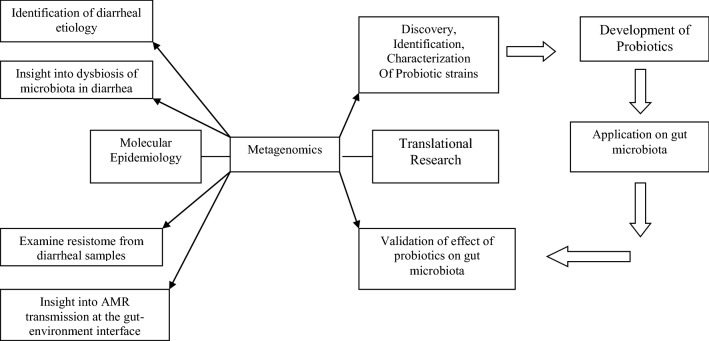
Application of metagenomics in assuaging the AMR problem in diarrhea

Effervescent metagenomic studies are being pursued globally by multiple groups which are helping in curating monumental information for the benefit of people to solve the problem of AMR through development of probiotics, alternative therapies and for intervention of AMR transmission network [67] (Fig. 2).

The enormous amount of data obtained with high-throughput method of metagenomics has generated the demand for highly advanced and precise computational analysis tools suitable to curate and annotate big data resulting in the development of highly efficient databases like MEGARes which are being extensively used for curating and analyzing antimicrobial resistance data augmenting global surveillance study outcomes on AMR. MEGARes is a hand-curated relational database and annotation structure. It is unique and superior to other available databases as it generates acyclic graphs (trees), annotates molecular function within sequence groups without disturbing nucleotide sequence identity [68].

## Conclusion

Metagenomics is expensive, labor-intensive, requiring sterling skills for wet-lab techniques, rigorous training for the operation of highly sophisticated instruments and expertise for analysis of high-throughput data of billions of sequence reads. Inspite of these stringent requirements it has intense and wide applicability.

It has helped in rapid characterization of taxonomically diverse members of the microbiota, discovery of organisms de novo, discovery of novel antibiotics and novel antibiotic synthesis pathways and in elucidation of new drug targets [48, 69]. It has provided significant insight into antibacterial action of bacterial proteins. These are examples of gigantic progress towards the development of alternative antimicrobial therapy [70]. Metagenomics has provided the basis for limitless perusal of intensive understanding of the physiological processes governing the microbiota [70]. It has helped in paving innumerable future possibilities to counteract AMR [71–73] and which are crucial for the prevention of diseases and for disease management [74]. It has emerged as an indispensible tool for the diagnosis of diarrhea [75]. It has helped scientists to sew up the gaps in molecular taxonomy and to recognize potential applications of prospective metagenomes. These historic milestones were achieved due to the potential of metagenomics in the redressal of previously unidentified microbes. It has helped to unfurl a world of unexplored microorganisms which had been neglected due to their non-culturability. Metagenomics is a versatile molecular tool which must be pursued with fervent endeavor for prognosis, diagnosis and commercial exploitation of microbiome for the prevention of emergence and spread of AMR globally.

## Data Availability

Data sharing is not applicable to this article as no datasets were generated or analysed during the current study.

## Abbreviations

AMR: antimicrobial resistance
ARGs: antimicrobial resistance genes
MGEs: mobile genetic elements
HGT: horizontal gene transfer
